# Long-term stability of recombinant tissue plasminogen activator at -80 C

**DOI:** 10.1186/1756-0500-2-117

**Published:** 2009-06-30

**Authors:** George J Shaw, Matthew Sperling, Jason M Meunier

**Affiliations:** 1 Department of Emergency Medicine, University of Cincinnati, Cincinnati, OH 45267-0769, USA; 2 Department of Biomedical Engineering, University of Cincinnati, Cincinnati, OH 45267-0769, USA

## Abstract

**Background:**

Recombinant tissue plasminogen activator (tPA) is a thrombolytic widely used clinically in the treatment of acute thrombotic disease such as ischemic stroke, myocardial infarction, and deep venous thrombosis. This has led to much interest in tPA based lytic therapies leading to laboratory based *in-vitro *and *in-vivo *investigations using this drug. However, tPA reconstituted in solution exhibits full activity for only 6–8 hours, according to the manufacturer. Therefore, methods to store reconstituted tPA for long durations while maintaining activity would be of assistance to laboratories using this enzyme.

**Findings:**

In this work, the enzymatic activity of tPA stored at -80 C over time was measured, using an ELISA technique that measured the amount of active tPA bound to plasminogen activator inhibitor 1 (PAI-1) in a given sample. Sample of tPA solution mixed to a concentration of 1 (mg/ml) were stored in cryogenic vials at -80 C for up to 7 years. For a given sample, aliquots were assayed for tPA activity, and compared with a tPA standard to determine relative enzymatic activity. Results are reported as means with standard errors, and 12 measurements were performed for each sample age.

**Conclusion:**

There was no decrease in tPA activity for samples stored up to 7 years. Such cryogenic storage is a viable method for the preservation of tPA solution for laboratory investigations of tPA-based lytic therapies.

## Findings

Recombinant tissue plasminogen activator (tPA) is widely to treat thrombotic disorders such as acute ischemic stroke, deep venous thrombosis [1]. As a result, there is much interest in optimizing lytic therapy using tPA in the treatment of such disease [2]. Such optimization requires substantial *in*-*vitro *and *in*-*vivo *[3] work. Therefore the preservation of tPA for laboratory use in such studies is of substantial interest to laboratories engaged in such research.

Currently, tPA is available as a lyophilized powder from the manufacturer (Genentech, San Francisco, CA). According to the manufacturer, tPA can be used for 8 hours after reconstitution in sterile water at room temperature; this is a short period of time for laboratory use. Therefore, methods to store reconstituted tPA for long durations while maintaining activity would be of assistance to laboratories using this medication. In this work, the enzymatic activity of tPA stored at -80 C over time was measured.

The tPA was obtained as a lyophilized powder and mixed with sterile water to a concentration of 1 mg/mL (580,000 IU/ml), as per the manufacturer's instructions. Solution samples were then stored at -80 C as 0.5–2 mL aliquots for 0.5 to 7.2 years in cryogenic vials. For activity measurements, samples were thawed at room temperature and diluted with 3% bovine serum albumin in tris buffered saline to achieve a tPA concentration of 0.36, 0.73, 1.45, 2.9 or 3.6 (IU/ml). Three measurements at a given concentration were performed for each sample. The samples were assayed for tPA activity using an ELISA technique that measures active tPA using plasminogen activator inhibitor 1 (PAI-1) (Human tPA Activity Assay, #IHTPAKT, Innovative Research, Novi, MI, USA). In this assay, a biotinylated human PAI-1 antibody binds to the active tPA in a given sample. Inactive tPA or tPA complexed with another protein will not be detected.

Samples of the tPA solution were added to wells on the microtiter plate after preparation with the PAI-1 antibody. In addition, a tPA standard (provided with the kit) was used to prepare a series of standard solutions of 0.345, 0.69, 1.38, 2.76, and 4.14 IU/ml. After incubation, anti-human tPA antibody was added to the PAI-1 bound tPA. Finally, anti-rabbit horseradish peroxidase (HRP) was added, and 450 nm absorbance was determined (SpectraMax M5 dual-monochromator microplate reader, Molecular Devices Corporation, Sunnyvale, CA) as a measure of enzymatic activity. The intra-assay precision was 9.8%, as stated by the manufacturer.

The absorbance values for the tPA standard solutions were used to construct a calibration curve of absorbance vs tPA concentration; fits to the data were performed using Softmax Pro software (Molecular Devices Corporation, Sunnyvale, CA). This calibration curve was used to determine tPA activity in the various sample solutions from the absorbance values. tPA activity is reported (in percent) as the measured activity referenced by the standard activity at the same tPA concentration; an activity of 100% means that the sample tPA activity was equal to that of the standard. Twelve measurements were done for each sample age.

**Figure 1:**
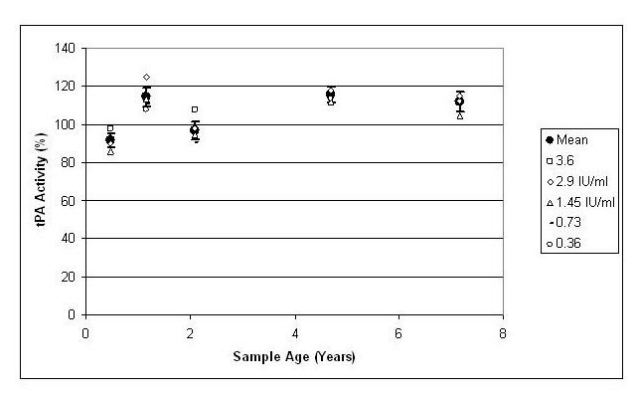
**tPA activity versus sample age for the data**. The solid circles denote the average activity for all samples at the given sample age, and the error bars are the 95% confidence limits. The individual data points are the mean tPA activity values for a given concentration; the values are shown in the legend. Overall, there is no decline in tPA activity over time.

Figure 1 shows tPA activity versus sample age. The closed circles with error bars are mean activity with 95% confidence limits for all measurements at that sample age. The individual data points are the mean activity at a given sample age for a given concentration of tPA, as explicated in the legend. There is no decrease of tPA activity with sample age, and there is no definite trend in tPA activity as a function of tPA concentration. These results are similar to work by others [4,5] for shorter durations. Overall, long-term low temperature storage of tPA preserves enzymatic activity and may be useful in preserving tPA solution for experimental use.

## Competing interests

The authors declare that they have no competing interests.

## Authors' contributions

GJS and JMM devised and planned the study, and GJS obtained the funding for the work. MS performed the tPA assays, and all authors assisted with data interpretation. GJS drafted the manuscript, and all authors assisted with revising the final paper, and approved the final document.
